# Trends and treatments of pelvic and acetabular fractures in Taiwan: facing an aging society

**DOI:** 10.1007/s11657-023-01255-5

**Published:** 2023-05-10

**Authors:** Shang-Lin Hsieh, Tsung-Li Lin, Yuan-Shun Lo, Chun-Yen Chen, Hao Wei Chang, Hsien-Te Chen, Yi-Chin Fong, Chun-Hao Tsai

**Affiliations:** 1 Department of Orthopedic Surgery, China Medical University Hospital, China Medical University, Taichung City, Taiwan; 2 Graduate Institute of Biomedical Sciences, China Medical University, Taichung, Taiwan; 3 Department of Sports Medicine, China Medical University, Taichung City, Taiwan; 4 Department of Orthopedic Surgery, China Medical University Bei Gang Hospital, Beigang, Yunlin County Taiwan; 5 Department of Orthopedic Surgery, Wei Gong Memorial Hospital, Toufen, Miaoli County Taiwan; 6 Department of Biomedical Engineering, College of Biomedical Engineering, Taichung, Taiwan

**Keywords:** Acetabular fracture, Aging, Epidemiology, Hip fracture, Incidence, National Health Insurance Research Database, Pelvic fracture

## Abstract

***Summary*:**

Pelvic-acetabular fractures lead to high mortality in elders and their association between different groups is not known. Our results indicate that older age with pelvic-acetabular fracture was significantly associated with mortality. This finding may help planning and allocating healthcare resources, risk stratification, and optimizing the treatment of pelvic fractures.

**Purpose:**

Pelvic or acetabular fractures are among main outcomes of low-energy trauma such as falls, especially in older adults. They represent approximately 3–8% of all fractures and are associated with a high mortality rate ranging from 4 to 28%. This study is aimed at comparing the incidence and trends of hip fractures and pelvic-acetabular fractures in the Taiwanese general population, gender differences in adults aged over 65 years, and mortality risk between pelvic or acetabular fractures and hip fractures and surgery trends in patients with these fractures.

**Methods:**

A retrospective study was conducted extracting data from the National Health Insurance Research Database of patients diagnosed with hip fracture and pelvic acetabular fracture between 2000 and 2018.

**Results:**

Older age with pelvic-acetabular fracture was significantly associated with increased mortality. No significant differences were found in comorbidities between the two fracture groups. Results provide clear epidemiological evidence for trends in pelvic-acetabular fractures in Taiwan and demonstrate the need for better strategies to manage these fractures and comorbidities, particularly in older adults.

**Conclusion:**

Findings of this study may aid in planning and allocating healthcare resources, risk stratification, and optimizing the treatment of pelvic fractures among older adults in Taiwan.

## Introduction

The hip joint is the largest weight-bearing joint in the human body, and it requires the coordinated movement of the pelvic bone, acetabulum, and femoral head to perform its essential functions. Due to the high levels of stress and impact that the hip joint experiences, bones in this region can fracture. While fractures in the pelvic bone, acetabulum, and femoral head can exhibit similar symptoms and be colloquially referred to as “hip fractures,” they are distinct fractures in the medical system. Fractures of the pelvic and acetabular bones occur in the pelvis and acetabulum, respectively. However, in orthopedics, the term “hip fracture” specifically refers to a fracture in the proximal femur. Understanding the differences between these types of fractures is essential for proper diagnosis and treatment.

Pelvic or acetabular fractures are rare injuries compared to fractures in other regions of the body, representing only about 3–8% of all fracture cases and associated with a high mortality rate of about 4–28% [1]. Pelvic fractures are one of the main results of low energy trauma such as falls, particularly in older adults. Similar to hip fractures, pelvic fractures are associated with high hospitalization rates, significant morbidity, and mortality and may lead to serious individual and socioeconomic burdens. Most patients with pelvic fractures die not from the pelvic fracture itself, but from the associated injury and decline in health status [2]. Pelvic fractures are underestimated osteoporotic or fragility fractures [3]. Compared to hip fractures, pelvic fractures have not yet been thoroughly investigated. A meta-analysis of 12 studies involving 5454 patients with pelvic fractures concluded that, in stable and alert trauma patients, a thorough clinical examination will detect pelvic fractures with a sensitivity of nearly 100% [4]. The diagnosis and differentiation of pelvic fractures with stable or unstable vital signs is particularly important from the perspective of medical management during emergency care prior to hospitalization. In addition, even if most pelvic fractures are not life-threatening, evaluating possible associated injuries is essential [5]. Comprehensive epidemiological surveillance of pelvic fractures in the presence of other injuries may provide more adequate information for pre-hospital responders and/or in-hospital staff.

Acetabular fractures, similar to pelvic fracture, are one of the most complex injuries in orthopedic medicine. Acetabular fractures mainly occur due to the impact of the femoral head on the articular surface. The fracture pattern depends on the position of the hip at the time of impact; for example, external rotation may lead to an anterior fracture pattern, and internal rotation may lead to a posterior fracture pattern [6]. Falls on the greater trochanter in older adults are likely to result in anterior column and/or wall fractures [7]. The studies of Letournel et al. [8, 9] increased the understanding among orthopedic specialists of surgical approaches for acetabular fractures, including reduction techniques, complications, and results. Good to excellent functional results have been reported in up to 80% of surgically treated acetabular fractures over 20 years [8, 9]. A variety of factors may influence clinical outcomes following acetabular fractures, including pre-existing conditions, injury-related factors, surgical considerations, and postoperative complications [6]. In addition, the quality of joint reduction is critical in determining the clinical outcomes. Age is clearly a risk factor for acetabular fractures; a previous study pointed to a marked increase in the incidence of acetabular fractures in older patients as the population ages [10].

Due to increasing in the older adult population globally, increased incidence of pelvic and acetabular fracture had already been reported in various geographic areas worldwide. This suggested that the burden of pelvic and acetabular fractures has become highly relevant for society in general and, in particular, for local and national healthcare systems. However, investigations regarding the incidence, mortality, and treatment trends of pelvic and acetabular fractures have primarily been conducted in Europe, including in Finland, Sweden, Germany, Austria, and France [11]. A previous study that reviewed 236 patients with pelvic fractures showed that 64.4% were injured in motor vehicle accidents, with a mean hospital stay of 16.8 days [12]. Another study including 128 women and 220 men with pelvic fractures also showed a mean hospital stay of 16.5 days [13]. But similar studies are not reported within the whole populations of Asian countries [14].

Taiwan is also facing an aging society just as, in Western countries, it is necessary to describe healthcare utilization and determine the burden of pelvic and acetabular fractures. The National Health Insurance (NHI) covers nearly all people in Taiwan, which is suitable for epidemiologic investigations [15]. Using evidence based on Taiwan’s national registry health data enables a more systematic investigation of the morbidity and mortality of in-hospital traumatic pelvic and acetabular fractures and the impact of major comorbidities. To figure out the incidence, mortality, and treatments between pelvic-acetabular fractures and hip fractures over time in a Taiwanese adult population, this retrospective study was conducted by analyzing patients’ data from NHI research database. We hypothesized that although the pelvic and acetabular fractures were similar to hip fracture in symptoms, they were led to different outcomes. This study should potentially aid in planning and allocating healthcare resources, risk stratification, and optimizing the treatment.

## Methods

### Study design and data source

This population-based, retrospective study extracted patient data from the National Health Insurance Research Database (NHIRD) of Taiwan, which contains comprehensive healthcare data, including sex, date of birth, employment, inpatient and outpatient diagnoses, procedures, surgeries, medication usage, and catastrophic illness, of approximately 23 million residents in Taiwan. The NHI Program of Taiwan, which was launched in 1995, provides universal and comprehensive healthcare coverage for approximately 99.9% of Taiwan residents [16] and diagnosis and procedure using the International Classification of Diseases, Ninth Revision, Clinical Modification (ICD-9-CM), and the International Classification of Diseases, Ninth Revision and Tenth Revision, Clinical Modification (ICD-9-CM and ICD-10-CM).

### Ethics statement

Because NHIRD consists of de-identified secondary data released to the public for research purposes, this study was exempt from full review by the IRB, and the informed consent of patients was waived. The study protocol was approved by the Institutional Review Board of China Medical University Hospital, Taiwan.

### Study population

The data of patients diagnosed with hip fracture and pelvic-acetabular fracture between 2000 and 2018 were extracted from the NHIRD. Inclusion criteria for patient selection were individuals who had been diagnosed with hip fracture (group 1) and pelvic-acetabular fracture (group 2) between the years 2000 and 2018. The index date was the date of first diagnosis of hip fracture or pelvic-acetabular fracture. Individuals younger than 65 years old and patients died or withdraw from the NHI before the index date were excluded. Four patients in group 1 were selected based on propensity score matching (PSM) with each patient in group 2, including age, sex, index year, and baseline comorbidities. Hip fractures were identified using ICD-9-CM: 820.0, 820.2, 733.14; ICD-10-CM: S72.019A, S72.023A, S72.033A, S72.043A, S72.099A, S72.109A, S72.143A, S72.23XA, and M84.459A. Pelvic and acetabular fractures were identified by ICD-9-CM: 808.0, 808.2, 808.4, and 808.8 and ICD-10-CM: S32.409A, S32.501A, S32.501A, S32.509A, S32.309A, S32.609A, S32.810A, S32.811A, S32.82XA, S32.89XA, and S32.9XXA.

### Main outcome and comorbidities

The primary endpoint of this study was all-cause mortality. All individuals were observed from the index date until the occurrence of death, withdrawal from NHIRD, or the end of follow-up (December 31, 2018), whichever came first. Regarding comorbid conditions, the most common risk factors that may affect fragility fractures were included such as Parkinson’s disease (ICD-9-CM: 332; ICD-10-CM: G20), end-stage renal disease (ESRD) (ICD-9-CM: 585; ICD-10-CM: N18), chronic obstructive pulmonary disease (COPD) (ICD-9-CM: 491, 492, 496; ICD-10-CM: J41-J44), stroke (ICD-9-CM: 430-438; ICD-10-CM: I60-I69), heart failure (ICD-9-CM: 428; ICD-10-CM: I50), coronary artery disease (CAD) (ICD-9-CM: 410-414; ICD-10-CM: I20.0, I20.1, I20.8, I20.9, I21. I22, I24.1, I24.8, I24.9, I25.1, I25.2), dementia (ICD-9-CM: 290, 294.1, 331.0-331.2; ICD-10-CM: F03.90), osteoporosis (ICD-9-CM: 733.0; ICD-10-CM: M81), and diabetes (ICD-9-CM: 250; ICD-10-CM: E08-E13).

### Statistical analysis

Categorical and continuous variables are shown as counts (percentage) and mean ± standard deviation (SD), respectively. Chi-square was used to evaluate between-group differences for categorical variables, and Student’s *t*-test was used to evaluate continuous data. Annual incidence rates of hip fracture and pelvic-acetabular fracture from 2000 to 2018 were calculated by sex in subjects older than 65 years. Crude (cHRs) and adjusted (aHRs) hazard ratios were calculated with confidence intervals (CIs) using univariable and multivariable Cox proportional hazard regression models to compare the mortality rates between hip fractures and pelvic-acetabular fractures. All statistical analyses were performed using SAS version 9.4 (SAS Institute Inc., Cary, NC, USA) software, and R software was used to draw the cumulative incidence curves by Kaplan-Meier method. A *p* value of less than 0.05 was regarded as statistical significance.

## Results

### Baseline characteristics of the study population

Table 1 summarizes the baseline demographic characteristics and comorbid conditions between patients with pelvic-acetabular fractures (*n* = 18,726) and hip fractures (*n* = 74,904). The proportion of males in the two groups was 32.13% (hip fracture) and 31.75% (pelvic-acetabular fracture), respectively. Most patients were aged between 65 and 79 years (65.50% of pelvic-acetabular fracture group) and (65.02% of hip fracture group). Mean ages of patients with hip fracture and pelvic-acetabular fractures were 76.63 and 76.47 years, respectively. All comorbidities compared between hip and pelvic-acetabular fracture were significant as the standardized mean difference (SMD) < 0.1. This included Parkinson’s disease, ESRD, COPD, stroke, heart failure, CAD, dementia, osteoporosis, and diabetes (Table 1).

**Table 1:** Patients’ baseline demographic characteristics and comorbidities between pelvic-acetabular fracture and hip fracture

	Hip fracture (*N* = 74904)		Pelvic-acetabular fracture (*N* = 18726)		
	*n*	%	*n*	%	SMD
Sex					
Female	50,838	67.87	12,780	68.25	0.008
Male	24,066	32.13	5946	31.75	0.008
Age (years)					
65–79	48,700	65.02	12,265	65.50	0.010
> 79	26,204	34.98	6461	34.50	0.010
Years, mean (SD)	76.63	(7.55)	76.47	(7.59)	0.020
Comorbidities					
Parkinson’s disease	3792	5.06	1163	6.21	0.050
ESRD	1514	2.02	557	2.97	0.061
COPD	28,113	37.53	7088	37.85	0.007
Stroke	26,510	35.39	6605	35.27	0.003
Heart failure	13,994	18.68	3683	19.67	0.025
CAD	34,346	45.85	8703	46.48	0.013
Dementia	7804	10.42	2007	10.72	0.010
Osteoporosis	29,759	39.73	7486	39.98	0.005
Diabetes	31,320	41.81	7850	41.92	0.002
Follow-up, years [mean (SD)]^†^	4.74	(4.19)	5.18	(4.48)	0.101

^†^Student’s *t*-test

*SMD*, standardized mean difference. A standardized mean difference of 0.1 or less indicates a negligible difference

*ESRD*, end-stage renal disease; *COPD*, chronic obstructive pulmonary disease; *CAD*, coronary artery disease

### Incidence of hip fractures and pelvic-acetabular fractures between 2000 and 2018 of age > 65 years old in Taiwan

**Fig. 1:**
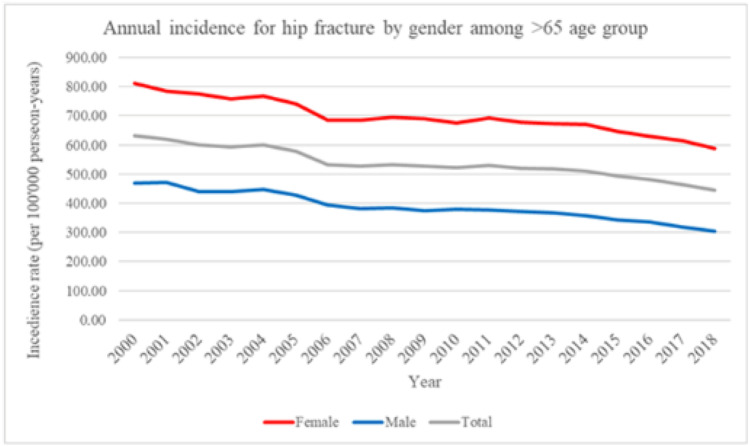
Temporal trends of hip fracture from 2000 to 2018 in Taiwan. Annual incidence of hip fracture from 2000 to 2018 in Taiwan. Among patients older than 65 years by gender

**Fig. 2:**
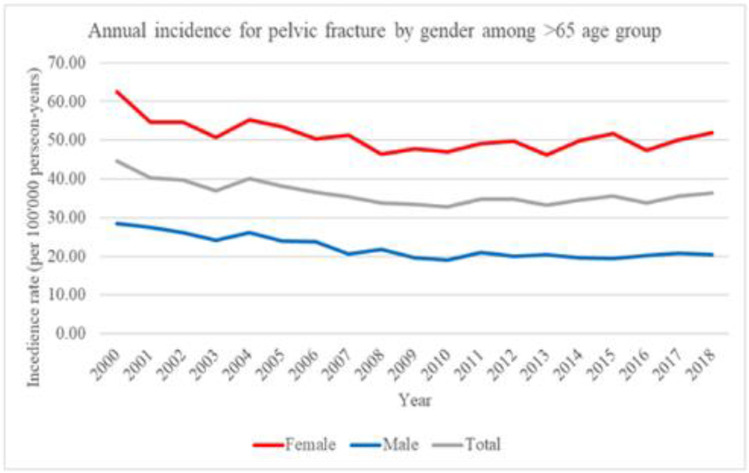
Temporal trends of pelvic-acetabular fracture from 2000 to 2018 in Taiwan. Annual incidence of pelvic-acetabular fracture from 2000 to 2018 in Taiwan. Among patients older than 65 years by gender

Figures 1 and 2 showed the temporal trends of hip fractures and pelvic-acetabular fractures in Taiwanese older than 65 between the years 2000 and 2018. Figure 1 showed the annual incidence of hip fractures among patients > 65 years by sex. Hip fracture incidence was higher in older females than that in males. For both males and females, hip fracture incidence gradually declines in older adults > 65 years of age.

Figure 2 showed incidence of pelvic-acetabular fractures among patients > 65 years, where women had twice incidence rate than the entire study period. Also, for both males and females, the incidence of pelvic-acetabular fracture gradually declined in older adults > 65 years.

### Cumulative incidence of death during follow-up between patients with hip fractures and pelvic-acetabular fractures of age > 65 years old

**Fig. 3:**
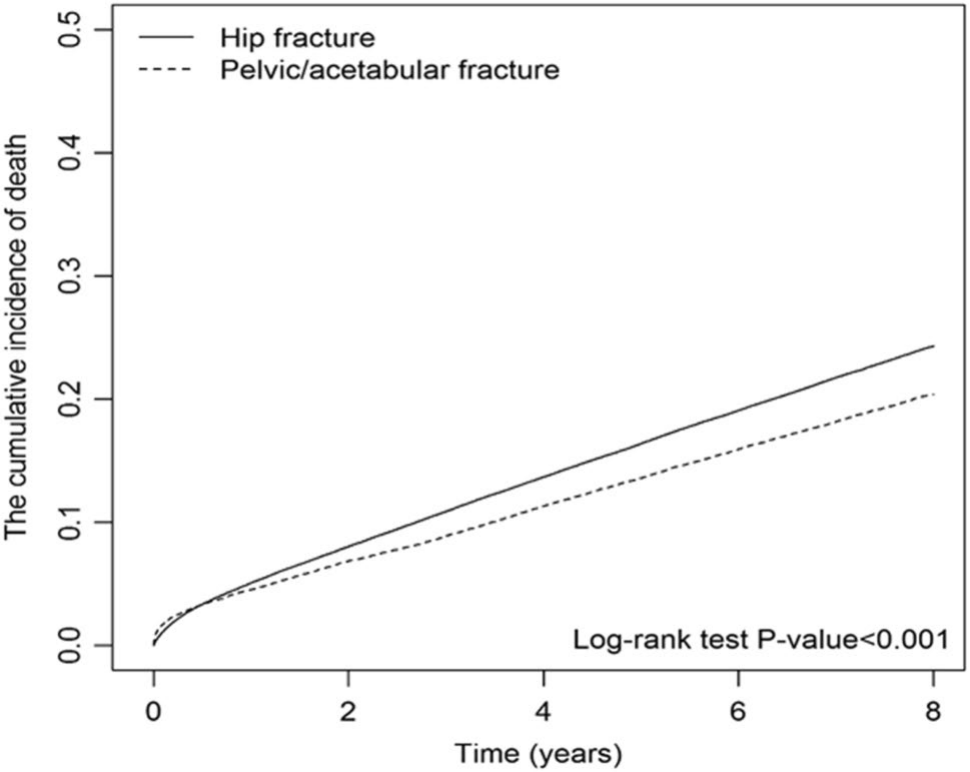
Cumulative incidence of death during follow-up period between patients with hip fractures and pelvic-acetabular fractures

Figure 3 shows the Kaplan-Meier survival curves by Cox proportional hazards analysis, indicating that patients with hip fractures had significantly higher cumulative incidence of mortality than those with pelvic-acetabular fractures (*p* < 0.001) (Fig. 3). Examination of the ten leading causes of death in the study cohort are summarized in Supplementary Table S1 and S2. In patients with pelvic-acetabular fractures, the top three causes of death were pneumonia, unspecified diabetes mellitus without complications, and multiple fractures involving both upper limbs and upper limb with rib(s) and sternum. In patients with hip fracture, the top three causes of deaths were pneumonia, unspecified DM without complications, and unspecified COPD.

### Risk factors of death in patients with pelvic-acetabular fracture and hip fracture of age > 65 years old

Table 2 summarizes the hazard ratios of death in the study population. After adjusting for confounders in the multivariable regression model, pelvic-acetabular fractures were associated with significantly lower risk of mortality than hip fractures (aHR, 0.82; 95% CI, 0.81–0.84). Male sex (aHR, 1.36; 95% CI, 1.33–1.39) and advanced age (aHR, 2.18; 95% CI, 2.14–2.22), on the other hand, were associated with significantly increased risk of mortality. Comorbidities associated with significantly increased risk of mortality included Parkinson’s disease (aHR, 1.12; 95% CI, 1.08–1.16), ESRD (aHR, 2.55; 95% CI, 2.42–2.69), COPD (aHR, 1.13; 95% CI, 1.11–1.15), stroke (aHR, 1.15; 95% CI, 1.13–1.17), heart failure (aHR, 1.40; 95% CI, 1.37–1.43), dementia (aHR, 1.27; 95% CI, 1.23–1.31), and diabetes (aHR, 1.22; 95% CI, 1.20–1.24). Osteoporosis, on the contrary, showed a significantly reduced risk or mortality in hip fracture than pelvic-acetabular fracture (aHR, 0.89; 95% CI, 0.87–0.91).

**Table 2:** Risk factors of death in patients with hip fractures and pelvic-acetabular fractures

	Death						
Variables	*n*	PY	IR	cHR	(95% CI)	aHR	(95% CI)
Fracture type							
Hip	41,318	355,051	116.37	1.00	(Reference)	1.00	(Reference)
Pelvic-acetabular	9449	96,933	97.48	0.84	(0.82, 0.86)***	0.82	(0.81, 0.84)***
Sex							
Female	32,611	313,316	104.08	1.00	(Reference)	1.00	(Reference)
Male	18,156	138,668	130.93	1.25	(1.23, 1.27)***	1.36	(1.33, 1.39)***
Age							
65–79	28,965	338,166	85.65	1.00	(Reference)	1.00	(Reference)
> 79	21,802	113,818	191.55	2.29	(2.25, 2.33)***	2.18	(2.14, 2.22)***
Comorbidities							
Parkinson’s disease	3068	19,044	161.10	1.46	(1.40, 1.51)***	1.12	(1.08, 1.16)***
ESRD	1507	5173	291.34	2.55	(2.42, 2.68)***	2.55	(2.42, 2.69)***
COPD	20,916	151,237	138.30	1.39	(1.37, 1.42)***	1.13	(1.11, 1.15)***
Stroke	19,404	137,679	140.94	1.41	(1.39, 1.44)***	1.15	(1.13, 1.17)***
Heart failure	11,686	65,084	179.55	1.77	(1.73, 1.81)***	1.40	(1.37, 1.43)***
CAD	24,178	188,036	128.58	1.28	(1.25, 1.30)***	0.99	(0.97, 1.01)
Dementia	6080	32,775	185.51	1.73	(1.68, 1.77)***	1.27	(1.23, 1.31)***
Osteoporosis	18,919	168,592	112.22	1.00	(0.98, 1.02)	0.89	(0.87, 0.91)***
Diabetes	21,070	164,366	128.19	1.24	(1.22, 1.26)***	1.22	(1.20, 1.24)***

*PY*, person-years; *IR*, incidence rate per 1000 person-years; *cHR*, crude hazard ratio; *aHR*, adjusted hazard ratio

^†^Adjusted by sex, age, and comorbidities

^*^p value < 0.05; **p < 0.01; ***p < 0.001

*ESRD*, end-stage renal disease; *COPD*, chronic obstructive pulmonary disease; *CAD*, coronary artery disease

### Risk of death in patients with pelvic-acetabular fracture versus hip fracture stratified by duration of follow-up of age > 65 years old

As shown in Table 3, Cox proportional-hazards regression model was used to compare the risk of the death in patients with pelvic-acetabular fractures versus hip fractures, stratified by different duration of follow-up. After adjusting for sex, age, and comorbidities, patients with pelvic-acetabular fractures had significantly lower risk of death among follow-up durations 3 to 6 years (aHR, 0.91; 95% CI, 0.87–0.96) and > 6 years (aHR, 0.79; 95% CI, 0.76–0.83) as compared to those with hip fractures.

**Table 3:** Risk of death in patients with pelvic-acetabular fracture versus hip fracture, stratified by follow-up duration

	Hip fracture			Pelvic-acetabular fracture						
Follow-up duration, years	*n*	PY	IR	*n*	PY	IR	cHR	(95% CI)	aHR	(95% CI)
< 3	20,153	166,444	121.08	4390	42,407	103.52	1.00	(0.97, 1.03)	1.00	(0.96, 1.03)
3–6	10,115	95,872	105.51	2309	26,131	88.36	0.93	(0.89, 0.97)**	0.91	(0.87, 0.96)***
> 6	11,050	92,736	119.16	2750	28,396	96.85	0.81	(0.78, 0.85)***	0.79	(0.76, 0.83)***

*PY*: person-years; *IR*: incidence rate per 1,000 person-years; *cHR* : crude hazard ratio; *aHR*: adjusted hazard ratio

†adjusted by sex, age, comorbidities

*p<0.05, **p<0.01, ***p<0.001

### Risk of death in pelvic-acetabular fracture versus hip fracture stratified by sex, age, and comorbidities

Table 4 summarizes the risk of death between pelvic-acetabular fracture versus hip fracture stratified by age, sex, and comorbidities. Compared with hip fracture, pelvic-acetabular fractures were associated with significantly lower mortality among males (aHR, 0.73; 95% CI, 0.70–0.76), females (aHR, 0.89; 95% CI, 0.86–0.91), patients aged 65–79 years (aHR, 0.76; 95% CI, 0.74–0.78), and above 79 years (aHR, 0.91; 95% CI, 0.88–0.94). Similarly, pelvic-acetabular fractures were significantly associated with lower mortality than hip fractures among patients with or without most comorbidities.

**Table 4:** Risk of death between pelvic-acetabular fracture and hip fracture patients stratified by sex, age, and comorbidities

	Hip fracture			Pelvic-acetabular fracture			Crude			Adjusted		
Variable	*n*	PY	IR	*n*	PY	IR	cHR	cCI	*p* value	aHR^†^	aCI	*p* value
Sex												
Female	26,349	247,966	106.26	6262	65,350	95.82	0.90	(0.88, 0.93)***	< 0.001	0.89	(0.86, 0.91)***	< 0.001
Male	14,969	107,085	139.79	3187	31,584	100.91	0.74	(0.71, 0.76)***	< 0.001	0.73	(0.70, 0.76)***	< 0.001
Age												
65–79	23,749	264,602	89.75	5216	73,564	70.90	0.79	(0.76, 0.81)***	< 0.001	0.76	(0.74, 0.78)***	< 0.001
> 79	17,569	90,449	194.24	4233	23,369	181.14	0.93	(0.90, 0.96)***	< 0.001	0.91	(0.88, 0.94)***	< 0.001
Comorbidities												
Parkinson’s disease												
No	38,928	340,630	114.28	8771	92,310	95.02	0.83	(0.81, 0.85)***	< 0.001	0.82	(0.80, 0.84)***	< 0.001
Yes	2390	14,421	165.73	678	4624	146.64	0.88	(0.81, 0.96)**	0.003	0.87	(0.8, 0.95)**	0.0016
ESRD												
No	40,211	351,315	114.46	9049	95,497	94.76	0.83	(0.81, 0.85)***	< 0.001	0.82	(0.8, 0.84)***	< 0.001
Yes	1107	3736	296.31	400	1437	278.43	0.95	(0.85, 1.06)	0.3686	0.94	(0.84, 1.06)	0.3048
COPD												
No	24,341	236,767	102.81	5510	63,979	86.12	0.84	(0.81, 0.86)***	< 0.001	0.82	(0.8, 0.85)***	< 0.001
Yes	16,977	118,283	143.53	3939	32,954	119.53	0.84	(0.81, 0.87)***	< 0.001	0.82	(0.8, 0.85)***	< 0.001
Stroke												
No	25,571	246,620	103.69	5792	67,685	85.57	0.83	(0.80, 0.85)***	< 0.001	0.81	(0.79, 0.83)***	< 0.001
Yes	15,747	108,431	145.23	3657	29248	125.03	0.86	(0.83, 0.89)***	< 0.001	0.84	(0.81, 0.87)***	< 0.001
Heart failure												
No	32,016	304,466	105.16	7065	82,434	85.70	0.81	(0.79, 0.84)***	< 0.001	0.81	(0.79, 0.83)***	< 0.001
Yes	9302	50,585	183.89	2384	14,499	164.43	0.90	(0.86, 0.94)***	< 0.001	0.88	(0.84, 0.92)***	< 0.001
CAD												
No	21,765	207,600	104.84	4824	56,348	85.61	0.82	(0.79, 0.84)***	< 0.001	0.81	(0.78, 0.83)***	< 0.001
Yes	19,553	147,451	132.61	4625	40,585	113.96	0.86	(0.83, 0.89)***	< 0.001	0.84	(0.82, 0.87)***	< 0.001
Dementia												
No	36,420	329,546	110.52	8267	89,664	92.20	0.84	(0.82, 0.86)***	< 0.001	0.82	(0.8, 0.84)***	< 0.001
Yes	4898	25,505	192.04	1182	7270	162.59	0.84	(0.79, 0.90)***	< 0.001	0.85	(0.79, 0.90)***	< 0.001
Osteoporosis												
No	26,038	221,809	117.39	5810	61,583	94.34	0.81	(0.79, 0.83)***	< 0.001	0.79	(0.77, 0.81)***	< 0.001
Yes	15,280	133,242	114.68	3639	35,350	102.94	0.90	(0.86, 0.93)***	< 0.001	0.89	(0.85, 0.92)***	< 0.001
Diabetes												
No	24,234	225,912	107.27	5463	61,707	88.53	0.83	(0.80, 0.85)***	< 0.001	0.81	(0.79, 0.84)***	< 0.001
Yes	17,084	129,139	132.29	3986	35,227	113.15	0.85	(0.82, 0.88)***	< 0.001	0.84	(0.81, 0.87)***	< 0.001

*PY*, person-years; *IR*, incidence rate per 1000 person-years; *cHR*, crude hazard ratio; *aHR*, adjusted hazard ratio

^†^Adjusted by sex, age, and comorbidities

^*^p value < 0.05; **p < 0.01, ***p < 0.001

*ESRD*, end-stage renal disease; *COPD*, chronic obstructive pulmonary disease; *CAD*, coronary artery disease

## Discussion

To date, no studies have compared the epidemiology of pelvic and acetabular fractures, including morbidity, mortality, and management, in the entire population of an Asian country. This study investigated and compared the incidence and trends of hip fractures and pelvic-acetabular fractures in the general population of adults aged over 65 years in Taiwan from 2000 to 2018. The study also assessed demographic and comorbid risk factors for death after pelvic-acetabular and hip fractures and the mortality rates between pelvic-acetabular and hip fractures. Study results showed that women had higher rates of pelvic-acetabular and hip fractures than men and both types of fracture occurred commonly in subjects aged 60 to 79 years. Individuals with pelvic-acetabular fractures were less likely to die than those with hip fractures. However, older age significantly increased mortality in patients with pelvis and hip fractures. Common comorbidities in the aging population such as COPD, CAD, stroke, and DM were all significantly associated with greater risk of death in pelvic-acetabular fractures and hip fractures as compared with no such comorbidities. In addition, we found that pneumonia was the leading cause of death for pelvic-acetabular fractures and hip fractures during follow-up.

More than 90% of hip fracture patients are over the age of 65 years and have comorbidities. Both of these factors have a strong impact on patients’ prognosis and treatment [17]. Although the annual incidence of pelvic-acetabular fractures and hip fractures appears to be increasing gradually, the incidence is decreasing in adults older than 65 years. Pelvic fractures are one of the major outcomes of low-energy trauma, such as falls, and are clinically associated with high hospitalization rates and high mortality similar to hip fractures [18]. The incidence of pelvic fractures has increased in various regions of the world due to the increasing global geriatric population, suggesting that the burden of pelvic fractures will be highly relevant to society as a whole, and particularly to our healthcare system. Current research on morbidity, mortality, and treatment trends for pelvic and acetabular fractures has mainly been conducted in certain countries in Europe. Similar to trends in Taiwan, the trends of pelvis and hip fracture incidence have been rising gradually in recent years. A previous study indicated that trends in the incidence of pelvic and femoral fractures varied widely in Sweden between 2001 and 2016 [19]. While the incidence of femoral fractures, including the hip, femoral shaft, and distal femur decreased or remained constant over the study year, the incidence of pelvic fractures increased. In that study, mortality rates varied between fractures, with the highest rate of death in patients with hip fractures [19].

Several studies in Germany also reported that the incidence of pelvic fractures increased with age. One previous study indicated that the incidence of pelvic fractures among older people in Germany was estimated to be even higher when compared to other countries [20]. In the same study population, increased mortality rates were found in the first several months after pelvic fracture, even after adjustment for sex, age, type of pelvic fracture, insurance, healthcare costs, comorbidities, and level of care. In Germany, pelvic fractures are projected to become increasingly relevant to society as a whole, especially the German healthcare system, due to demographic changes and the aging population worldwide [21]. Other previous studies have indicated that the number of osteoporotic pelvic fractures in Finland is growing faster than the aging population and that effective prevention is urgently needed to control these age-related increases in fractures [22]. The same study showed that between 1970 and 2002, a marked increase was seen in the number and incidence of low-trauma pelvic fracture admissions in Finnish women aged 80 years or older. Another study also noted that the annual number of pelvic ring fractures among older people in Finland is increasing at a rate that cannot be explained by demographic changes alone [23]. For general fractures, effective preventive measures are needed to control the occurrence of fractures including focusing on reducing risk factors such as bone loss, falls, and fractures in older adults who are prone to falls.

An Austrian study reported that patients aged 65 and older with pelvic fractures had a higher risk of death [24]. In terms of fracture rates, Austria has one of the highest rates of hip fractures and distal forearm fractures globally. Furthermore, the observed number of pelvic fractures was even higher than expected. Similar to Taiwan, Austria has a social health insurance system that covers 99.9% of the country’s population. It has one of the lowest self-reported unmet medical needs in the European Union, but Austria still spends far more on hospitalization than most countries. A previous French study clarified that the incidence of acetabular and pelvic fractures is increasing rapidly, especially in older adults, with a substantial increase expected by 2030 [23]. In addition, treatment is increasingly resorting to surgery. Public health strategies are needed to reduce morbidity and improve treatment. Further research is needed to determine the best strategy, as there is currently no consensus on treatment, especially in the older adult population.

Taiwan has also entered the aging society as in Western countries. It is necessary to describe the use of healthcare to determine the burden of pelvic fractures. According to global trends, the incidence of pelvic-acetabular fractures in the older adult population is expected to double in the next 20 years [6]. The past two decades have greatly contributed to the understanding of pelvic-acetabular fracture morphology, biomechanics, associated comorbidities, and principles of fracture fixation, providing the perfect foundation for the development of this subspecialty. The advent of different types of plates for specific fractures, the advent of newer surgical approaches, the use of therapeutic agents to prevent intraoperative blood loss, and advances in radio-diagnosis have also had a dramatic impact on the management outcomes of these complex injuries. However, real-world evidence from the developing world is relatively scarce.

The present study reports the temporal trends of pelvic-acetabular and hip fractures in Taiwan during the past 20 years, as well as associated trends of mortality. Pelvic-acetabular fractures have a high mortality rate, and older age in this study was significantly associated with increased mortality. This study was the first report to assess trends in pelvic-acetabular fractures over 20 years in Taiwan, and results may provide clear epidemiological evidence for trends in pelvic-acetabular fractures in Taiwan. Results also demonstrate the need for better strategies by which to manage these fractures and comorbidities in older adults. Since the number of pelvic-acetabular fractures and hip fractures is increasing, and advanced age significantly affects the prognosis of these fractures, results of the present study have certain clinical implications. In particular, the mortality rate for both types of fractures is high, highlighting the importance of optimal treatment. As surgery emerges as the treatment of choice, suggesting that geriatric-appropriate surgery and better postoperative care must be developed for this specific patient subgroup. The findings of this study will aid in planning and allocating healthcare resources, risk stratification, and optimizing the treatment of pelvic fractures.

## Limitations

The present study gained strength from the large population-based database but the retrospective nature of the study has certain inherent limitations, including that result cannot be generalized to other populations or locations and follow-up data for each patient is limited, which limits long-term evaluation. The NHI database did not provide information on the actual severity of fractures such as severity assessed by injury severity score (ISS) using the diagnostic code system. We also had no information about each patient’s lifestyle factors such as exercise level or daily activity, which may have added insight into risk factors and causes of fracture.

## Conclusions

The present study is the first to report an assessment of the trends of pelvic-acetabular fractures in Taiwan over a 65-year period. The incidence and mortality of these two fractures are high in Taiwan, and older age and comorbidities are significantly associated with increased mortality in the study population. The results emphasize the need to develop better strategies for both preventing and managing these fractures among older adults.

## Data Availability

The dataset used in this study is held by the Taiwan Ministry of Health and Welfare (MOHW). The Ministry of Health and Welfare must approve our application to access this data. Any researcher interested in accessing this dataset can submit an application form to the Ministry of Health and Welfare requesting access. Please contact the staff of MOHW (Email: stcarolwu@mohw.gov.tw) for further assistance. Taiwan Ministry of Health and Welfare Address: No.488, Sec. 6, Zhongxiao E. Rd., Nangang Dist., Taipei City 115, Taiwan (ROC). Phone: +886-2-8590-6848.

## Declarations

### Disclaimer

The funders had no role in the study design, data collection and analysis, the decision to publish, or preparation of the manuscript.

## References

[1] Harrison A, Ordas-Bayon A, Chimutengwende-Gordon M, Fortune M, Chou D, Hull P, Carrothers A, Rawal (2022). Factors associated with mortality in older patients sustaining pelvic or acetabular fractures. Arch Orthop Trauma Surg.

[2] Zhao W, Zhao J, Liu T, Liu Z, Liu L, Zhang Y (2022). Incidence and risk factors of preoperative deep venous thrombosis following pelvic and acetabular fractures: a retrospective case-control study. J Orthop Surg Res.

[3] Rommens PM, Hofmann A (2021). Focus on fragility fractures of the pelvis. Eur J Trauma Emerg Surg.

[4] Sauerland S, Bouillon B, Rixen D, Raum MR, Koy T, Neugebauer EA (2004). The reliability of clinical examination in detecting pelvic fractures in blunt trauma patients: a meta-analysis. Arch Orthop Trauma Surg.

[5] Marrinan S, Pearce MS, Jiang XY, Waters S, Shanshal Y (2015). Admission for osteoporotic pelvic fractures and predictors of length of hospital stay, mortality and loss of independence. Age Ageing.

[6] Trikha V, Tornetta P (2020). Management of pelvi-acetabular injuries: global scenario and future trends. J Clin Orthop Trauma.

[7] Court-Brown CM, Clement ND, Duckworth AD, Biant LC, McQueen M (2017). The changing epidemiology of fall-related fractures in adults. Injury.

[8] Letournel E (1993) The treatment of acetabular fractures through the ilioinguinal approach. Clin Orthop Relat Res 292:62–76 8519138

[9] Letournel E (1993) Open treatment of acute calcaneal fractures. Clin Orthop Relat Res 290:60–67 8472472

[10] Navarre P, Gabbe BJ, Griffin XL, Russ MK, Bucknill AT, Edwards E, Esser MP (2020). Outcomes following operatively managed acetabular fractures in patients aged 60 years and older. Bone Joint J.

[11] Giannoudis PV, Pohlemann T, Bircher M (2007). Pelvic and acetabular surgery within Europe: the need for the co-ordination of treatment concepts. Injury..

[12] Poole GV, Ward EF, Muakkassa FF, Hsu HS, Griswold JA, Rhodes RS (1991) Pelvic fracture from major blunt trauma. Outcome is determined by associated injuries. Ann Surg 213(6):532–538 10.1097/00000658-199106000-00002 PMC1358569 2039283

[13] Poole GV, Ward EF (1994). Causes of mortality in patients with pelvic fractures. Orthopedics.

[14] Rommens PM, Hofmann A (2013) Comprehensive classification of fragility fractures of the pelvic ring: recommendations for surgical treatment. Injury, pp 1733–1744 10.1016/j.injury.2013.06.023 23871193

[15] Zhang XS, Leu FY, Yang CW, Lai LS (2018). Healthcare-based on cloud electrocardiogram system: a medical center experience in middle Taiwan. J Med Syst.

[16] Hsing AW, Ioannidis JP (2015). Nationwide population science: lessons from the Taiwan National Health Insurance Research Database. JAMA Intern Med.

[17] Menzies IB, Mendelson DA, Kates SL, Friedman SM (2012). The impact of comorbidity on perioperative outcomes of hip fractures in a geriatric fracture model. Geriatr Orthop Surg Rehabil.

[18] Eggenberger E, Hildebrand G, Vang S, Ly A, Ward C (2019). Use of CT Vs. MRI for diagnosis of hip or pelvic fractures in elderly patients after low energy trauma. Iowa Orthop J.

[19] Lundin N, Huttunen TT, Enocson A, Marcano AI, Felländer-Tsai L, Berg HE (2021). Epidemiology and mortality of pelvic and femur fractures-a nationwide register study of 417,840 fractures in Sweden across 16 years: diverging trends for potentially lethal fractures. Acta Orthop.

[20] Andrich S, Haastert B, Neuhaus E, Frommholz K, Arend W, Ohmann C, Grebe J, Vogt A, Brunoni C, Jungbluth P, Thelen S, Dintsios CM, Windolf J, Icks A (2021). Health care utilization and excess costs after pelvic fractures among older people in Germany. Osteoporos Int.

[21] Andrich S, Haastert B, Neuhaus E, Neidert K, Arend W, Ohmann C, Grebe J, Vogt A, Jungbluth P, Rösler G, Windolf J, Icks A (2015). Epidemiology of pelvic fractures in Germany: considerably high incidence rates among older people. PLoS One.

[22] Parkkari J, Kannus P, Niemi S, Pasanen M, Järvinen M, Lüthje P, Vuori I (1996). Secular trends in osteoporotic pelvic fractures in Finland: number and incidence of fractures in 1970–1991 and prediction for the future. Calcif Tissue Int.

[23] Kannus P, Parkkari J, Niemi S, Sievanen H (2015). Low-trauma pelvic fractures in elderly Finns in 1970–2013. Calcif Tissue Int.

[24] Behanova M, Haschka J, Reichardt B, Dimai HP, Resch H, Zwerina J, Kocijan R (2022) Pelvic fractures-an underestimated problem? Incidence and mortality risk after pelvic fracture in Austria, 2010-2018. J Clin Med 11(10):2834 10.3390/jcm11102834 PMC9146576 35628960

